# The Shortage of Subjects for Anatomical Dissection

**Published:** 1920-02-21

**Authors:** 


### ANATOMY ACT REFORM.

### I.—The Shortage of Subjects for Anatomical Dissection.

At the present time there is a serious shortage of subjects for anatomical dissection, and the trend of current events foreshadows some more or less radical alteration of the Anatomy Act, which, after much debate in both Houses of Parliament, was finally passed in the House of Lords during the year 1832.

Taking a retrospective view throughout the ages of the troubled history of dissection, one is greatly impressed by the antagonistic attitude of the general public, and this attitude, chiefly caused by ignorance, unless modified by suitable and lucid explanation, will in all probability prevent any great change in the existing Anatomy Act.

In the present article we shall devote ourselves entirely to the difficulties and drawbacks of the past. Great reforms are urgently necessary; and in a second article, after recent consultation with some of the leading surgeons and anatomists in London, we shall attempt to outline both the possibilities and probabilities ahead. The present sketch is perhaps in places almost unpleasant reading, and the atrocities of the long past are happily in no sense represented to-day; but one feels that the whole situation should be dealt with, or else left alone.

In the early part of the last century a reason which greatly added to the dislike of dissection amongst the public was that it was regarded as a disgrace, for it was one of the penalties attached to capital punishment. All felons who had died by the hands of the law were handed over to the anatomist, and very often some official from the Royal College of Surgeons proceeded to Newgate in order to claim the body. When the first Anatomy Bill was passed the clause for the dissection of criminals was retained; but, owing to the strong objections of the leading surgeons and anatomists, the clause was removed. This sagacious manœuvre, which did much to improve the public's opinion, had an interesting historic precedent; for Pope Benedict XIV. of Bologna, a man devoted to science, issued a decree in the early part of the eighteenth century commanding that all patients dying in the Bologna hospitals should be dissected; at the same time, in his wisdom, Pope Benedict decreed that dissection was no longer to be made one of the penalties attaching to capital punishment.

### The "Morning Herald" of 1831.

Any scientific measure, even of the greatest public value, has always been vigorously opposed by some sections of the community, and the Anatomy Act was no exception to this rule. A very good example of such cavilling we quote from a leading article entitled "The Anatomy or Dead-body Bill," which was published in the *Morning Herald* at the time the Act was being placed before the House of Lords by the first Duke of Wellington.

"Putting aside for the present the question as to the generous and noble propriety of the Government of the country taking advantage of the Unfortunate, and cruelly adding to the dreadful misery of the friendless and forlorn wretch who dies in a workhouse or hospital, the appalling prospect of having his body after death (a period which all look forward to as the season of rest) consigned to be mangled and torn by the knife of the ruthless anatomist, is far from being popular amongst the medical profession, for whose benefit and that of science it is so artfully said to be intended."

### Protagonists of Dissection.

In the endeavour to overcome the prejudices against dissection, instances are recorded of persons leaving their bodies to the anatomist for subsequent dissection. The most celebrated case in recent years was that of Jeremy Bentham, who made a will in 1769 bequeathing his body for dissection, but the old gentleman did not die until 1832, at the ripe age of eighty-two years. After his death his body was taken to the Webb Street School of Anatomy, and a lecture delivered over his remains by the principal of the institution. In 1788 a Dr. Monsey, who was attached to the Chelsea Hospital, made a will directing that his body should not be "insulted by any funeral ceremony," but should undergo dissection. In accordance with the will, a Mr. Forster, surgeon, of Broad Street, City, dissected the body and delivered a lecture on it to the students of Guy's Hospital.

### "The Resurrectionists."

The prevailing mode of securing bodies prior to the passing of the present Act was deplorable in the extreme. Numbers of men recruited from the lowest of the community were employed in supplying these bodies as a regular trade to the teachers of anatomy, and their usual source of supply was the London churchyards and the private burial-grounds in the poorer parts of the great city.

The remuneration of the "Resurrectionists" was not very liberal; as much as six guineas per subject was sometimes obtained, but the prevailing figure was three and a-half guineas, although in Dublin they could be got for twenty-five shillings, and sometimes ran as low as ten. A light cart was the usual mode of transit, but sometimes a Hackney coach would be used for the same purpose.

In 1829, at Edinburgh, two criminals named Burke and Hare were convicted of murdering more than a dozen people for the sake of selling their bodies. Sir W. Fergusson was demonstrator of anatomy at Edinburgh at the time these atrocities were committed. This event so shocked the public that a Royal Commission was immediately appointed by the Government, and from their report the regulations of the Anatomy Act were framed.

From this time onward the terrible proceedings of the past entirely ceased, and for many years now the provisions then made have worked satisfactorily, but their period of efficiency is also now finished, and reform we must have.

The Committee advised that we should work on the plan adopted by our present Allies, the French—viz., that all persons dying in hospitals or infirmaries, if not claimed twenty-four hours after death, may be sent for dissection.

### Will History Repeat Itself?

There were some eight hundred medical students in London at the time of the Royal Commission, and of these scarcely five hundred were able to dissect; in consequence, between two and three hundred students carried out their dissections in Paris.

At the present time the supply of subjects is quite inadequate for teaching purposes in England. Will our students once again emigrate to Paris, or will our Government supply this definite need with suitable legislation? These are at least possibilities open to them, as we will endeavour to show in the next article.

